# Cold-Induced Physiological and Biochemical Alternations and Proteomic Insight into the Response of *Saccharum spontaneum* to Low Temperature

**DOI:** 10.3390/ijms232214244

**Published:** 2022-11-17

**Authors:** Bao-Qing Zhang, Yu-Xin Huang, Zhong-Feng Zhou, Shan Zhou, Wei-Xing Duan, Cui-Fang Yang, Yi-Jing Gao, Ge-Min Zhang, Xiu-Peng Song, Xiao-Qiu Zhang, Ao-Mei Li, Dong-Liang Huang, Yang-Rui Li

**Affiliations:** Key Laboratory of Sugarcane Biotechnology and Genetic Improvement (Guangxi), Ministry of Agriculture and Rural Affairs/Guangxi Key Laboratory of Sugarcane Genetic Improvement/Sugarcane Research Institute, Guangxi Academy of Agricultural Sciences/Sugarcane Research Center, Chinese Academy of Agricultural Sciences, Nanning 530007, China

**Keywords:** sugarcane, *Saccharum spontaneum*, cold tolerance, physiological and biochemical indices, proteomics, differentially abundant proteins (DAPs)

## Abstract

Sugarcane, a cash crop, is easily affected by low temperature, which results in a decrease in yield and sugar production. Breeding a new variety with cold tolerance is an essential strategy to reduce loss from cold stress. The identification of germplasms and genes/proteins with cold tolerance is a vital step in breeding sugarcane varieties with cold tolerance via a conventional program and molecular technology. In this study, the physiological and biochemical indices of 22 genotypes of *S. spontaneum* were measured, and the membership function analysis method was used to comprehensively evaluate the cold tolerance ability of these genotypes. The physiological and biochemical indices of these *S. spontaneum* genotypes showed a sophisticated response to low temperature. On the basis of the physiological and chemical indices, the genotypes were classified into different cold tolerance groups. Then, the high-tolerance genotype 1027 and the low-tolerance genotype 3217 were selected for DIA-based proteomic analysis by subjecting them to low temperature. From the four comparison groups, 1123, 1341, 751, and 1693 differentially abundant proteins (DAPs) were identified, respectively. The DAPs based on genotypes or treatments participated in distinct metabolic pathways. Through detailed analysis of the DAPs, some proteins related to protein homeostasis, carbohydrate and energy metabolism, amino acid transport and metabolism, signal transduction, and the cytoskeleton may be involved in sugarcane tolerance to cold stress. Furthermore, five important proteins related to cold tolerance were discovered for the first time in this study. This work not only provides the germplasms and candidate target proteins for breeding sugarcane varieties with cold tolerance via a conventional program and molecular breeding, but also helps to accelerate the determination of the molecular mechanism underlying cold tolerance in sugarcane.

## 1. Introduction

Sugarcane (*Saccharum* spp.) is an important sugar and energy crop across the world, mainly distributed in tropical and subtropical regions [1]. Plants face variable environmental conditions during their growth. Abiotic stresses such as cold, drought, and flood severely impact the growth and development of plants [2]. These stresses can cause cell metabolism disorders and produce a series of morphological, physiological, and molecular changes, leading to reduced crop yields or even no production [3]. As with other crops, low temperature is one of the main limiting factors for the growth, yield, and quality of sugarcane [4]. China is one of the main countries for sugarcane production in the world. Guangxi, the main sugarcane producer in China, often suffers from severe cold damage. For instance, the cold weather in 2008 caused a direct economic loss of 4.6 billion CNY in sugarcane production in Guangxi, and the impacted sugarcane accounted for 32.2% of the total sugarcane growing areas in Guangxi [5].

Cold or low temperature induces the accumulation of reactive oxygen species (ROS) in cells, and directly leads to cell oxidative damage, physiological metabolism disorders, and impaired plant growth, quality, and yield in crops [6,7,8,9,10]. In addition to the decrease in photosynthetic rate and leaf transpiration rate, the sucrose content also decreases in roots, followed by leakage of the root sap due to alterations in cell membrane permeability under freezing conditions in sugarcane [11]. Water infiltration due to rapid freezing/thawing can also lead to softening of the root tissue and gradual rotting [12].

To adapt to unfavorable low temperature environments, plants have gradually evolved complex mechanisms to avoid and tolerate cold stress. Cold tolerance in apple is associated with increased ethylene content, and further studies revealed that cold-induced ethylene release may be associated with MdERF1B, which positively regulates cold tolerance and ethylene biosynthesis in an MDCiBHLH1-dependent manner [13]. Plant hormones are involved in the cold stress response of *Zanthoxylum bungeanum* Maxim by regulating carbon, fatty acid, amino acid, and glucose metabolism, as well as other metabolic pathways [14]. C-repeat-binding factors (CBFs) play a key role in plant cold tolerance by increasing COR gene expression. Two U-box type E3 ubiquitin ligases, PUB25 and PUB26, degrade MYB15, a transcriptional repressor of CBF-dependent signaling, and increase CBF expression under cold stress, thereby enhancing *Arabidopsis* cold tolerance [15]. Under low-temperature stress, the chloroplast and mitochondrial structures of the sugarcane genotypes with high ploidy were more severely damaged than genotypes with low ploidy. Correlation analysis of the morphological indices and cold tolerance revealed a significant negative correlation between cold tolerance and ploidy [16]. Transcriptome analysis of sugarcane exposed to cold stress showed that cytoderm-related genes played a crucial regulatory role in sugarcane response to cold stress, and sugarcane miRNA was involved in sugarcane cold stress response [17,18,19]. Comparative genomic analysis of sugarcane exposed to cold stress revealed that ScADH3, a gene belonging to the ethanol dehydrogenase family that regulates ROS to maintain ROS homeostasis in sugarcane, is related to sugarcane cold tolerance [20]. In response to cold stress, some physiological and biochemical indices change dramatically in plants, mainly including reactive oxygen species (ROS), superoxide dismutase (SOD), catalase (CAT), peroxidase (POD), malondialdehyde (MDA), soluble protein (SP), proline (Pro), and soluble sugar (SS) [7,21,22,23]. By comparing these physiological and biochemical indicators, the cold-tolerant ability of the germplasms can be evaluated.

Breeding cold-tolerant sugarcane varieties is a key strategy to reduce loss from cold stress. Identification of germplasms with cold tolerance is a vital step to breed sugarcane varieties with cold tolerance via a conventional breeding program. Moreover, molecular breeding has been successfully used in improving some important traits in many crops [24]. Hence, molecular breeding is an effective way to improve cold tolerance in sugarcane varieties. Identification of the key genes controlling cold tolerance in sugarcane is a prerequisite to improving the cold tolerance of sugarcane varieties using molecular technology. Proteomic analysis can screen out proteins related to some important traits [25]. It has been used in sugarcane to identify key proteins related to sucrose accumulation [26,27], disease response [28,29,30], and drought stress [31]. However, proteome analysis regarding cold tolerance has not yet been investigated in sugarcane.

*Saccharum spontaneum*, the main source of tolerance genes in sugarcane, is one of the most valuable germplasms for sugarcane breeding [32,33]. In this study, the cold tolerance ability of 22 genotypes of *S. spontaneum* was evaluated as a function of physiological and biochemical indicators and classified into different groups on the basis of their cold tolerance ability. Then, two genotypes with contrasting cold tolerance ability were selected for further proteomic analysis. Some proteins responding to cold stress, which may be the key factors regulating cold tolerance, were identified. This work not only provides the germplasms for breeding sugarcane varieties with cold tolerance via a conventional program but also provides candidate targets for improvement of the cold tolerance in sugarcane via molecular breeding, which can hopefully accelerate the determination of the molecular mechanism underlying cold tolerance in sugarcane.

## 2. Results

### 2.1. S. spontaneum Genotypes Show Sophisticated Physiological and Biochemical Response to Low Temperature

The physiological and biochemical parameters of the 22 tested genotypes of *S. spontaneum* were compared under room temperature (25 °C) and low temperature (4 °C) conditions at different timepoints to better understand the non-enzymatic and enzymatic responses of *S. spontaneum* to low temperature.

#### 2.1.1. Soluble Protein (SP)

**Figure 1:**
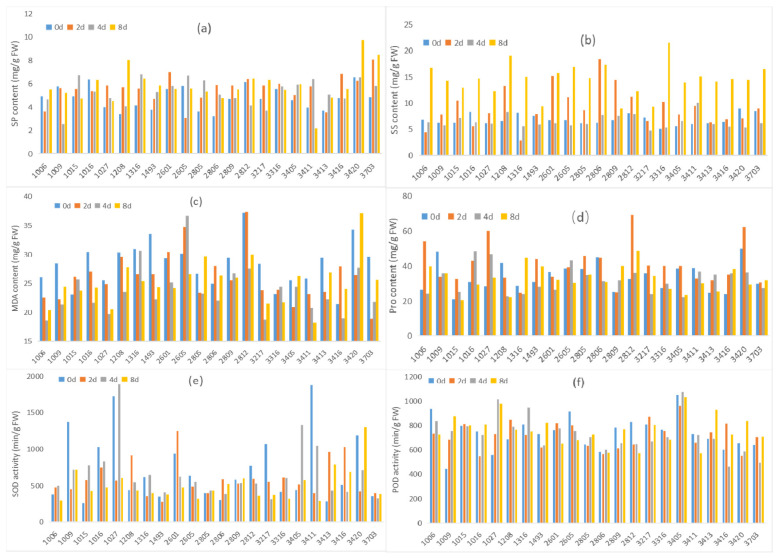
Physiological and biochemical response of 22 genotypes of *Saccharum spontaneum* to low-temperature stress. (**a**) SP, (**b**) SS, (**c**) MDA, (**d**) Pro, (**e**) SOD, and (**f**) POD. Note: According to the measurement results, the value of each parameter showed no significant variation in each control sample at different sampling points; therefore, only the value at 0 days is shown in the figure for clarity.

After 2, 4, and 8 days of low-temperature treatments, the SP content in genotypes 1027, 1028, 1316, 1493, 2805, 2806, 2809, 3405, and 3703 was higher than that in the room-temperature treatment at the three timepoints. The SP content in 1015 and 3411 gradually increased on the second day and fourth days of stress treatment and was higher than that in the control; however, as the stress continued, on the eighth day, the SP content decreased and was lower than that of the control group. After the low-temperature treatment, the SP content in genotypes 1006, 3413, and 3420 decreased but increased again up to the eighth day as compared with control. The SP content in genotypes 2812, 3217, and 3416 showed a trend of increasing, then decreasing, and finally increasing, being higher than that in the control on the eighth day. The SP content in genotypes 1009 and 1016 was always lower than that in the control, while that in genotypes 2601 and 3316 increased on the second day, and then gradually decreased with the extension of stress time, finishing lower than that in the control. The SP content in genotype 2605 was lower than that in the control at most timepoints except for a significant increase on the fourth day of treatment (Figure 1a).

#### 2.1.2. Soluble Sugar (SS)

After low-temperature treatment, the change trend of SS content in the leaves of each *S. spontaneum* genotype could be divided into four categories. Genotypes 1006, 1016, and 1316 showed a decreasing–increasing–increasing trend; genotypes 3217 and 3420 decreased before increasing, and exceeded the control on the eighth day. The SS content in genotype 3411 increased throughout under low-temperature stress. The remaining 16 genotypes showed an increasing–decreasing–increasing trend. It should be noted that, after low-temperature treatment, except for the fourth day, the SS content in each genotype generally increased, becoming very obvious on the eighth day. Only genotype 3411 maintained an increase throughout (Figure 1b).

#### 2.1.3. Malondialdehyde (MDA)

Low temperature induced the accumulation of MDA in the leaves of each genotype to varying degrees; however, only that in genotype 1015 was always higher than that in the control. The MDA content in genotypes 2805, 2806, 3405, 3416, and 3420 showed a trend of decreasing or increasing on the second or fourth day to eighth day of low-temperature treatment, which finished higher than that in the control. Although the MDA content in genotypes 2605, 2601, 2812, and 3316 was higher than that in the control on the second or fourth day, it showed a later and lower decrease than the control. The MDA content in the other 12 genotypes was always lower than that in the control (Figure 1c).

#### 2.1.4. Proline (Pro)

The Pro content in leaves of genotypes 1009, 1208, 2601, 2806, and 3411 decreased to a value lower than that in the control after low-temperature treatment at different timepoints. However, that in genotypes 1005, 1006,1016, 1316, 2605, 2805, 3217, 3316, 3405, and 3420 ended up lower than that in the control on the eighth day after undergoing an increase or decrease on the second or fourth day of treatment. The opposite trend was shown for genotypes 1493, 2809, and 3703, whose Pro content increased significantly, finishing higher than that in the control on the eighth day of low-temperature stress. The Pro content in genotypes 1027, 2812, 3413, and 3416 always exceeded than that in control. It is worth noting that the Pro content in genotypes 1006, 1027, 2812, and 3420 increased sharply on the second day, and then decreased significantly, albeit remaining higher that in the control except for genotype 3420 on the eighth day of treatment (Figure 1d).

#### 2.1.5. Superoxide Dismutase (SOD)

Low temperature inhibited the SOD activity in genotypes 1009, 1016, 1027, 3217, 3411, and 3420, whereby only genotype 3420 could gradually recover and exceed the SOD activity recorded in the control on the eighth day. The SOD activity in genotype 3411 recovered somewhat on the fourth day compared to the second day, but it was lower than that in the control. The SOD activity in all treatments and genotypes reached the highest level on the second day, except for genotype 2601, which showed a higher level on the eighth day at low temperature and was higher than that in the control (Figure 1e).

#### 2.1.6. Peroxidase (POD)

Genotypes 1009, 1027, 1208, and 3413 had higher POD activity than the control. In particular, the POD activity in genotypes 1009 and 1027 was higher than that in the control from the second day onward. Genotypes 1015, 1016, 1493, 2805, 3416, 3420, and 3703 also had the ability to maintain or restore POD activity in the leaves, and they showed higher POD activity than the control on the eighth day of stress (Figure 1f).

### 2.2. The S. spontaneum Genotypes Could Be Classed into Several Groups with Different Cold Tolerance on the Basis of Physiological and Biochemical Indices

**Figure 2:**
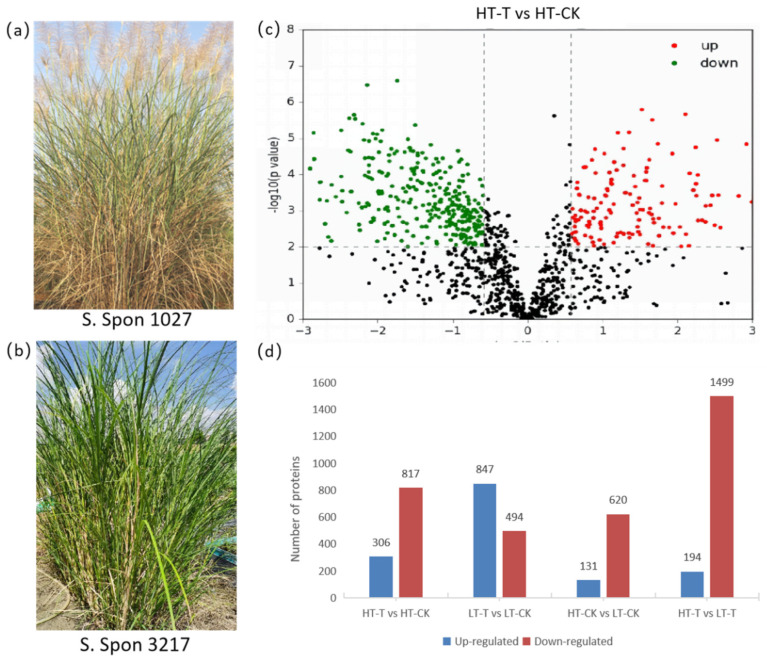
Phenotypes of two *S. spontaneum* genotypes for proteomics analysis and differentially abundant proteins from all comparison groups. (**a**) Phenotype of *S. spontaneum* 1027 with high tolerance to cold. (**b**) Phenotype of *S. spontaneum* 3217 with low tolerance to cold. (**c**) Volcano map of differentially abundant proteins from HT-T vs. HT-CK comparison. Red dots indicate upregulated (multiple of variation > 1.2, *p* < 0.05) proteins, green dots indicate downregulated (multiple of variation < 0.833, *p* < 0.05) proteins, and black dots indicate non-differentially expressed proteins. (**d**) Comparison of DAPs based on genotypes and treatments. Change ratio = 1.5 and *p* = 0.01 were selected as the differential protein threshold.

The cold tolerance of the 22 S. *spontaneum* genotypes under low-temperature treatment was evaluated using the membership function value method. The membership function values included SP, SS, MDA, Pro, SOD, and POD, and the results are shown in Table 1. According to the average membership function values, the cold tolerance ability of 22 *S. spontaneum* genotypes was divided into three levels: high tolerance (HT), medium tolerance (MT), and low tolerance (LT). Among the 22 genotypes, only two genotypes, 1027 and 3411, were classed as high-tolerance, while four genotypes were classed as low-tolerance, among which genotype 3217 had the weakest cold tolerance. According to these results, high-tolerance genotype 1027 (Figure 2a) and low-tolerance genotype 3217 (Figure 2b) were selected as the tested materials for proteomic analysis.

### 2.3. Low-Temperature-Induced Proteomic Alternations Revealed by DIA-Based Strategy

To identify the protein response to low temperature, the two genotypes with contrasting tolerance ability were subjected to low temperature for 8 days, with room temperature as a control. Samples were collected for DIA-based quantitative proteomic analysis. The samples were designated as HT-T (high-cold-tolerance genotype treated with low temperature), LT-T (low-cold-tolerance genotype treated with low temperature), HT-CK (high-cold-tolerance genotype treated with room temperature), and LT-CK (low-cold-tolerance genotype treated with room temperature). A total of 2816 proteins were identified in this study (Table S1), and 1123 differentially abundant proteins (DAPs) were obtained from the high-tolerance genotype comparing cold stress and control (HT-T vs. HT-CK), with 306 upregulated and 817 downregulated proteins (Figure 2c,d; Table S2). In the low-tolerance genotype comparison (LT-T vs. LT-CK), there were 1341 differential abundant proteins, of which 847 were upregulated and 494 were downregulated (Figure 2d; Table S3). Regarding the comparison between genotypes, there were 751 DAPs (HT-CK vs. LT-CK) under normal-temperature conditions, which was the lowest among the four comparison groups, of which 131 were upregulated and 620 were downregulated (Figure 2d; Table S4). After low-temperature treatment, 1693 DAPs were obtained between genotypes (HT-T vs. LT-T), which was the largest number among the four comparison groups, of which 194 were upregulated and 1499 were downregulated (Figure 2d; Table S5).

### 2.4. Distinct Metabolic Pathways Were Involved in the Response of S. spontaneum to Low Temperature

**Figure 3:**
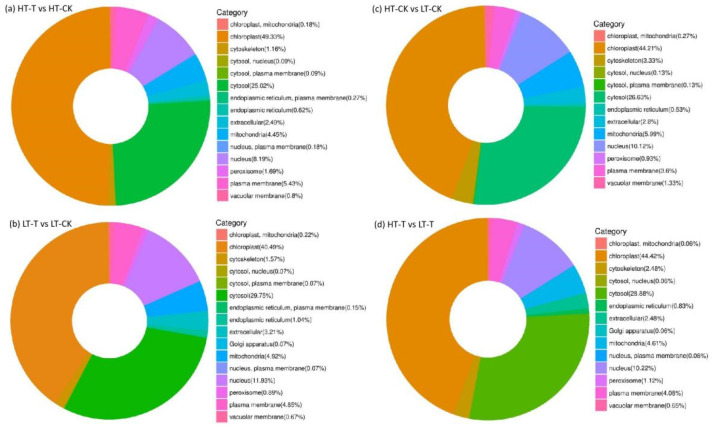
The subcellular localization of differentially abundant proteins from all comparison groups.

To clarify the response of different genotypes to low temperature, we performed a statistical analysis on the DAPs from the four comparison groups. According to subcellular localization analysis, the DAPs in all comparisons were mainly localized in the chloroplasts (> 40%), followed by cytosol (> 25%), cell nucleus (> 8%), and mitochondria (about 5%). However, the percentage of each category differed across comparisons (Figure 3).

**Figure 4:**
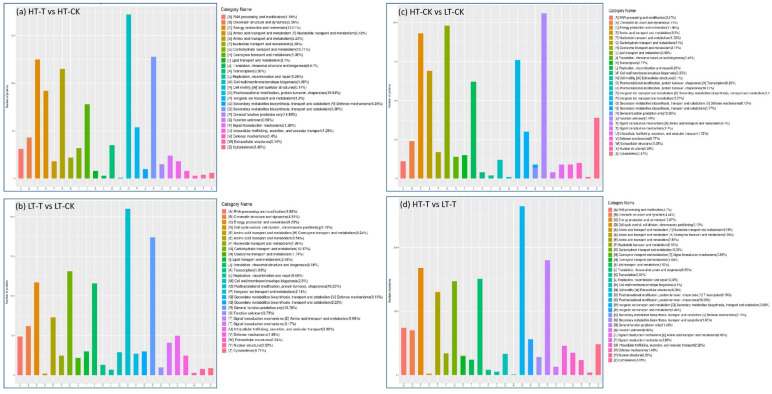
COG functional classification of the differentially abundant proteins. The abscissa is the COG category, while the ordinate is the number of differentially abundant proteins in each category.

Further COG analysis on the DAPs from the four comparisons showed that the DAPs from all four groups were divided into 24 categories. The six COG categories O, R, C, G, E, and J were the largest, mainly related to post-translational modification, protein conversion, and molecular chaperones (O), general function prediction (R), energy production and conversion (C), carbohydrate transport and metabolism (G), amino acid transport and metabolism (E), and translation, ribosome structure, and biogenesis (J). Among them, categories O and R accounted for the highest proportions in the HT-T vs. HT-CK (Figure 4a; Table S6), LT-T vs. LT-CK (Figure 4b; Table S7), and HT-T vs. LT-T (Figure 4d; Table S8) comparison groups, while the higher proportions were recorded for categories R and G in the HT-CK vs. HT-CK comparison group (Figure 4c; Table S9). The percentage of each category differed across comparison groups. These results confirmed the distinct mechanisms via which different genotypes respond to low temperature.

### 2.5. Identification of Protein Response to Cold Stress in Sugarcane

Considering their response to low temperature, the genes differentially expressed between genotypes with different cold tolerance may also be involved in cold tolerance in sugarcane; hence, to identify the genes/proteins from the pathways related to cold tolerance in sugarcane, we performed KEGG analysis on the DAPs from all four comparisons.

**Figure 5:**
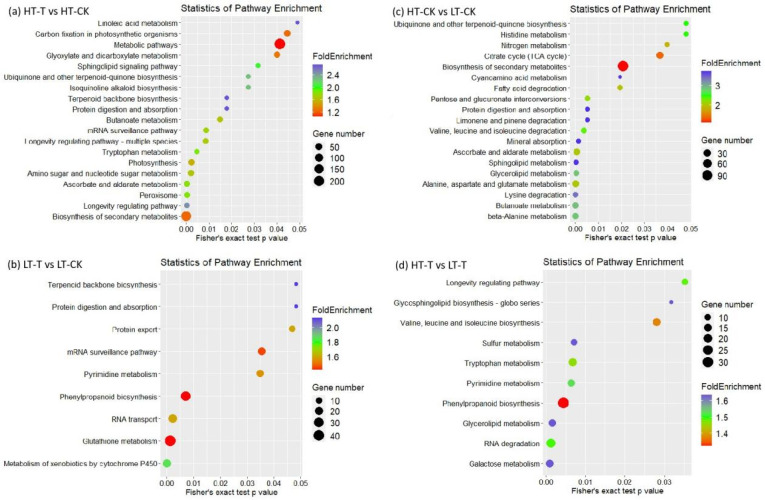
KEGG pathway analysis on differentially abundant proteins from each comparison. Colors indicate the degree of enrichment. Blue represents stronger enrichment, green represents strong enrichment, and red represents moderate enrichment. The *p*-value according to Fisher’s exact test indicates the significance level of the difference.

The DAPs from high-tolerance genotype *S. spontaneum* 1027 between treatment and control (HT-T vs. HT-CK) were highly enriched in the biosynthesis of secondary metabolites, ascorbate and aldarate metabolism, peroxisome, amino sugar and nucleotide sugar metabolism, photosynthesis, tryptophan metabolism, etc. (Figure 5a; Table S10). DAPs from low-tolerance genotype *S. spontaneum* 3217 before and after treatment (LT-T vs. LT-CK) were highly enriched in cytochrome P450-mediated metabolism of xenobiotics, glutathione metabolism, RNA transport, phenylpropanoid biosynthesis, pyrimidine metabolism, etc. (Figure 5b; Table S11). The results indicate that DAPs from the two genotypes with contrasting cold tolerance participated in distinct pathways, which may be the main reason for the tolerance difference between genotypes.

To further explore the possible metabolic pathways related to cold tolerance from the angle of difference between genotypes, we also conducted KEGG analysis on comparisons between genotypes. The DAPs between genotypes without treatment (HT-CK vs. LT-CK) were highly enriched in β-alanine metabolism, butanoate metabolism, lysine degradation, alanine, aspartate and glutamate metabolism, etc. (Figure 5c; Table S12). The DAPs between genotypes after treatment (HT-T vs. LTT) were enriched in galactose metabolism, RNA degradation, glycerolipid metabolism, phenylpropanoid biosynthesis, and tryptophan metabolism (Figure 5d; Table S13).

To accurately identify the protein response to cold stress in sugarcane, we further analyzed the abundance alteration of the proteins in the KEGG pathways. For instance, in the comparison of the high-tolerance genotype between cold stress and control (HT-T vs. HT-CK), in the ubiquitination-mediated protein degradation pathway (Figure S1), the proteins involved in the biosynthesis of ubiquinone and other terpene quinones were more active, and NAD(P)H dehydrogenase (quinone) [EC:1.6.5.2] was upregulated, while MPBQ/MSBQ methyltransferase [EC:2.1.1.295] and tocopherol cyclase [EC:5.5.1.24] were downregulated. In the photosynthesis-related pathway (Figure S2), photosystem II, photosystem II oxygen-evolving enhancer protein 1(PsbO), photosystem II oxygen-evolving enhancer protein 2 (PsbP), photosystem II oxygen-evolving enhancer protein 3 (PsbQ), and photosystem II 22 kDa protein (PsbS) were downregulated. In photosystem I, photosystem I subunit X (PsaK) was upregulated, while photosystem I subunit IV (PsaE) and three other proteins (PsaF, PsaL, PsaN) were downregulated. Cytochrome b6 (Pet B) in the cytochrome b6/f complex was downregulated, ferredoxin (Pet F) in the photosynthetic electron transport pathway was upregulated, and plastocyanin (Pet E) was downregulated. F-type ATPase including F-type H^+^-transporting ATPase subunit gamma (ATPF1G, atpG) and F-type H^+^-transporting ATPase subunit delta (ATPF1D, atpH) were downregulated. In addition, among the proteins involved in amino sugar and nucleotide sugar metabolism (Figure S3), alpha-l-arabinofuranosidase [EC:3.2.1.55], reversibly glycosylated polypeptide/UDP-arabinopyranose mutase [EC:5.4.99.30], and UDPglucose 6-dehydrogenase [EC:1.1.1.22] were upregulated, whereas GDP-d-mannose 3′,5′-epimerase [EC:5.1.3.18], phosphoglucomutase [EC:5.4.2.2], and glucose-1-phosphate adenylyltransferase [EC:2.7.7.27] were downregulated.

Moreover, some important proteins in pathways related to phenylpropanoid biosynthesis, peroxisome, tryptophan metabolism, biosynthesis of secondary metabolites, alanine, aspartate, and glutamate metabolism, and pentose and glucuronate interconversions also showed significant variation in comparisons based on genotypes or treatment (Figure 5; Tables S10–S13). These proteins may also be involved in sugarcane tolerance to cold stress.

### 2.6. PRM Verification of Five Proteins Induced by Low Temperature

In order to test the accuracy of the DIA proteomic analysis results in this study, the abundances of five proteins, Sspon.01G0025850-1A, Sspon.07G0016410-2P, Sspon.04G0017410-4P, Sspon.06G0005070-1A, and Sspon.03G0012410-2B, which may play a critical role in the cold tolerance of *S. spontaneum*, were tested using PRM. The abundances of these proteins in different comparisons tested using PRM were consistent with those derived from DIA analysis, confirming the reliability of the proteomic analysis in this study (Table 2).

## 3. Discussion

The sugarcane and sugar industry has become the pillar industry for poverty alleviation and rural revitalization in the main sugarcane growing regions, such as Guangxi and Yunnan in China. Low temperature is one of the key unfavorable environmental factors limiting the stability and development of sugarcane production. Breeding new varieties with cold tolerance is an essential strategy to reduce the sugarcane production loss caused by low temperature. Identification of germplasms and genes/proteins with cold tolerance can accelerate the breeding of new varieties with cold tolerance via a conventional program and molecular technology.

Physiological and biochemical metabolism involves several aspects of plasma membrane permeability, osmotic regulation, and membrane lipid peroxidation [7,21,22,23,34]. When plants are subjected to adverse stress, the reactive oxygen species (ROS) in plants increase sharply; the main source of production is the electron transport chain in the mitochondria and chloroplasts. When ROS accumulates to a certain amount, it has a destructive effect on the cell, especially the membrane system [22]. Plants have a series of ROS-removal mechanisms to maintain the dynamic balance of ROS. There are two types of active oxygen scavenging mechanisms in plants: enzymatic and nonenzymatic. The enzymatic systems mainly include SOD, CAT, and POD. The nonenzymatic mechanisms involve substances such as SP, Pro, and SS, which are important osmotic regulators, and their accumulation can confer higher stress tolerance onto plants, such as cold tolerance [7] and drought tolerance [35]. MDA content is considered to be negatively correlated with the cold tolerance of plants, which can reflect the degree of damage to the plant membrane system [21,23]. In this study, low temperature induced changes in antioxidant enzyme activity and osmotic regulator content, among which SOD activity changed the most (Figure 1e). The difference in SS content in *S. spontaneum* between the control group and the low-temperature treatment group was significant (Figure 1b). In particular, the SS content on the eighth day was significantly higher than that of the control, suggesting that soluble sugars play a key role in protecting cells from damage under low-temperature stress. The MDA content in most genotypes decreased after low-temperature stress (Figure 1c), suggesting that it is negatively correlated with low temperature.

Previous research proved that membership function value can be effectively used in the evaluation of the important traits of a population The membership function value (MFV) method has been used to screen soybean genotypes for drought tolerance [36], to evaluate the leaf abscission sensitivity of cotton triggered by thidiazuron [37], and to determine mutton quality characteristics of Dongxiang Tribute Sheep [38]. Some physiological and biochemical indices, including ROS, SOD, CAT, POD, MDA, SP, Pro, and SS, in plants have been proven to respond to cold stress [7,21,22,23]. Hence, in this work, we used the membership function value method to evaluate the *S. spontaneum* genotypes on the basis of these physiological and biochemical indices. The 22 genotypes were classified into three groups with high tolerance (HT), medium tolerance (MT), and low tolerance (LT) to cold stress. At different time points of low-temperature stress, HT genotype 1027 always showed a higher SS content and SOD activity than LT genotype 3217. The SP content in HT genotype 1027 was higher than that in LT genotype 3217 on the second and fourth days of stress treatment, but it was lower than that in genotype 3217 on the eighth day. Although the Pro content in HT genotype 1027 was slightly lower than that in LT genotype 3217 on the eighth day of the treatment, it was significantly higher than that in LT genotype 3217 on the second and fourth days, indicating that HT genotype 1027 had a stronger membrane stability maintenance ability under low-temperature stress. Although the POD activity in HT genotype 1027 was lower than that in LT genotype 3217 on the second day of stress treatment, it was higher on the fourth and eighth days, while the POD activity under low-temperature stress was significantly higher than that in the control. These results support the notion that the cold tolerance of genotype 1027 to low-temperature stress was stronger than that of genotype 3217, and the degree of damage to the membrane system in genotype 1027 was lighter. These findings also validate the reliability of the MFV method and these indices for the evaluation of cold tolerance in sugarcane germplasms. Therefore, genotype 1027 and genotype 3217 with the most contrasting cold tolerances were selected for further proteomic analysis to identify cold-tolerant proteins/genes.

Identification of differentially abundant proteins can provide insights into the distinct metabolic pathways involved in the response of *S. spontaneum* to low temperature in sugarcane. According to bioinformatics analysis, the proteins related to protein homeostasis, carbohydrate and energy metabolism, amino acid transport and metabolism, signal transduction, and the cytoskeleton may be involved in sugarcane’s tolerance to cold stress.

### 3.1. Protein Homeostasis

Through proteomic analysis, we can understand the dynamic regulation state of key proteins in cells [39], i.e., protein turnover. In this study, some metabolic processes or pathways involved in protein synthesis or degradation, such as ribosomes, post-translational modifications, and RNA transport, were identified through DIA-based quantitative proteomic technology (Figure 4). Ribosomal protein was considered a key factor to resist cold stress [40,41]. Ribosomes are also important regulatory sites for gene expression in response to cold stress. STCH4 is a ribosome biogenesis factor that accumulates under cold conditions [42]. In this study, the expression levels of proteins involved in translation, ribosomal structure, and biogenesis function were different in the two genotypes of *S. spontaneum*; it is worth mentioning that four proteins, Sspon.07G0009240-3P, Sspon.02G0008140-3D, Sspon.07G0009240-4P, and Sspon.07G0009240-1A in genotype 1027 were significantly upregulated under low-temperature stress in contrast to genotype 3217. Thus, these proteins may be involved in sugarcane’s response to low-temperature stress.

Post-translational modifications are involved in almost all cellular pathways and processes, such as ubiquitination. Previous studies have shown that ubiquitin is also involved in the response of plants to stress [43]. NAD(P)H dehydrogenase can reduce the oxidative damage of tobacco leaves caused by high-temperature stress [44]. NDH-mediated PSI cycle electron transfer or chloroplast respiration under low-temperature condition provides additional energy for the carbon assimilation process by generating ATP [45]. These results indicate that NAD(P)H dehydrogenase can protect plants under unfavorable temperature conditions. Vitamin E (Vit E), i.e., tocopherol, plays a role in scavenging free radicals and protecting the integrity of membranes in the plant [46,47,48,49]. In this study, by annotating the biosynthetic pathways of ubiquinone and other terpene quinones of the highly tolerant genotype 1027, it was found that NAD(P)H dehydrogenase (quinone), MPBQ/MSBQ transmethylase, and tocopherol were upregulated, while cyclase was down-regulated (Figure S3; Tables S2 and S3), hinting these proteins may be involved in the response of plants to low-temperature stress.

RNA transport is an important biological process, RNA molecules can be introduced into organelles such as peroxisomes, mitochondria, and plastids [50]. There were six DAPs involved in RNA transport in this study (Sspon.07G0009240-3P, Sspon.07G0009240-4D, Sspon.02G0008140-3D, Sspon.02G0008140-1A, Sspon.07G0009240-4P, and Sspon.07G0009240-1A) (Table S1). After low-temperature treatment, these proteins were downregulated in genotypes 1027 and 3217; however, the downregulation in genotype 3217 was significantly higher than that in genotype 1027 (Tables S2 and S3), suggesting that these proteins related to RNA transport might be involved in the cold tolerance of *S. spontaneum*.

Heat-shock proteins are an important class of defensive proteins. The use of heat-shock proteins to stabilize proteins is considered to be the main mechanism of plant cold tolerance under low-temperature conditions [51]. Among all heat-shock protein families, only HSP70s (I32, I33, and I34) are upregulated in the cold stress response and eventually stabilize the protein conformation in *Physcomitrella patens* [52]. In this study, we found that 21 DAPs in the two *S. spontaneum* genotypes belonged to the heat-shock protein 70 family (Table S1). Among them, only two proteins were downregulated, while the other 19 were upregulated under low-temperature stress. Compared with the control plants, the upregulated expression of these proteins in genotype 1027 was lower than that in genotype 3217 (Tables S2 and S3). Hence, the increased expression of heat-shock proteins may be related to the increase in the damage effect under low-temperature stress, which helps the plant to enhance its self-protection ability under stress.

### 3.2. Carbohydrate and Energy Metabolism

Carbohydrates are the main components of the cell structure, with the function of providing energy and regulating cell activities during plant growth and development, such as participating in the plant response to low-temperature stress [53], which is mainly produced by the photosynthesis of plants. In this study, after sub-cellular structural positioning of all the differentially abundant proteins, it was found that the most localized position of these proteins at the subcellular level was the chloroplast (Figure 3), indicating that the chloroplast is the organelle significantly affected by low-temperature stress in the cells of *S. spontaneum*. In addition, there were 115 DAPs involved in carbohydrate transport and metabolism, as well as 125 DAPs involved in energy production and conversion, under low-temperature stress compared with the control, and most of these proteins were downregulated in genotype 1027 (Tables S2 and S6). In contrast, most proteins were upregulated in genotype 3217 (Tables S3 and S7), which suggests that low-temperature stress might inhibit the production of carbohydrates and energy in genotype 1027 compared to genotype 3217.

### 3.3. Amino Acid Transport and Metabolism

The accumulation of specific amino acids and secondary metabolites produced by amino acid metabolism is related to the increased tolerance of plants to adverse environmental conditions, such as abiotic stress [54], and the amino acid transport process is mediated by amino acid transporters. In this study, 92 and 70 proteins were found related to amino acid transport and metabolism in the two genotypes after low-temperature stress (Tables S6 and S7). Compared with the control, the upregulated expression of these proteins in genotype 1027 was higher than that in genotype 3217 (Tables S2 and S3), indicating they were involved in cold stress response. Plant growth and development are easily affected by low temperature. Low-temperature stress reduces plant photosynthesis, hinders energy production and substance synthesis, consumes energy, and increases hunger in plants [55]. This may be one of the reasons why the carbohydrate and energy production in genotype 1027 was further suppressed, despite a higher cold tolerance than genotype 3217.

### 3.4. Signal Transduction

In the functional classification of DAPs, compared with genotype 3217, three DAPs (Sspon.01G0049620-1P, Sspon.01G0049620-1B, and Sspon.01G0049620-2D) involved in signal transduction mechanisms were found to be obviously upregulated in genotype 1027 (Tables S4 and S5). They were enriched in starch and sucrose metabolism, mutual conversion of pentose and glucuronic acid, and ascorbic acid and aldonic acid metabolism pathways. The activation of starch metabolism enzymes can improve the cold adaptation of bananas, and sucrose has been shown to be necessary for its cryoprotection [56]. Der Agopian et al. [57] speculated that starch/sucrose metabolism may be part of the cold tolerance mechanism of banana fruits. Transcriptome and metabonomic analyses have indicated that starch and sucrose metabolism play a crucial role in the cold stress adaptation of rapeseed [58]. Metabolism is the general term for life-sustaining activities. Plants can withstand cold weather mainly by regulating their level of energy metabolism [6]. In this study, energy metabolism was accompanied by nitrogen metabolism, secondary metabolism, carbohydrate metabolism, and the metabolism of various amino acids. Plants adjust their physiological state under low-temperature stress to a new dynamic balance through these metabolic activities [59].

In addition, the cytoskeleton represents a very important category. Its network system includes microtubules, intermediate filaments, and actin filaments. A recent study showed that overexpression of a sugarcane tubulin gene *SoTUA* can significantly improve the cold tolerance of sugarcane [60]. In *Arabidopsis thaliana* leaves, the transcription level of TUB8 was regulated in response to low-temperature stress [61]. In addition, the carboxyl terminal of β-tubulin is also involved in the regulation of microtubule stability at low temperature in corn [62]. Among the 2816 proteins identified in this study, the proteins involved in cytoskeletal function mainly existed in the tubulin domain (18) and actin domain (33) (Table S1). Among them, the 16 proteins in the tubulin domain were all upregulated by low temperature in both high- and low-tolerance genotypes (Tables S2 and S3), which indicates that these proteins were involved in the response of *S. spontaneum* to low-temperature stress. In addition, it is worth mentioning that the content of tubulin in the leaves of low-cold-tolerance *Triticum aestivum* varieties was higher than that in high-cold-tolerance varieties [63]. The same result was obtained in this study, whereby the abundance level of tubulin protein in the leaves of the high-tolerance genotype 1027 was significantly lower than that in the low-tolerance genotype 3217 under low-temperature stress (Table S5). Actin can induce the expression of cold response genes [64], and actin has been shown to play a positive role in the cold tolerance process of *Arabidopsis* [65]. Among the 2816 proteins identified in this study, 22 and 11 actin family members were upregulated in the HT-T vs. HT-CK (Table S2) and LT-T vs. LT-CK (Table S3) comparison groups. This suggests that more actin proteins were involved in the cold stress response in the high-tolerance genotype, and it is very likely that actin provides a greater contribution to the cold tolerance of *S. spontaneum*. Otherwise, the ratio of tubulin/actin determines the low-temperature stability of microtubules in the tissue [63]. In this study, after comparing the two genotypes under low-temperature conditions (HT -T vs. LT-T), it was found that the expression level of actin in genotype 1027 was almost significantly lower than that in genotype 3217 (Table S5). On the other hand, the tubulin/actin ratio in genotype 1027 was also lower than that in genotype 3217. This low rate would lead to a high cold tolerance of cells [63,66], which is consistent with the results of the high tolerance of genotype 1027 and the low tolerance of genotype 3217, while also highlighting the importance of actin in cold tolerance and the ability of actin to maintain the stability of the cytoskeleton of *S. spontaneum* [61].

Lastly, we propose five important proteins related to the cold tolerance of *S. spontaneum*, discovered for the first time in this study: Sspon.01G0025850-1A, Sspon.07G0016410-2P, Sspon.04G0017410-4P, Sspon.06G0005070-1A, and Sspon.03G0012410-2B. Sspon.01G0025850-1A contains a canonical TOG domain that regulates microtubule dynamics and is involved in intracellular vesicle trafficking. It has been shown that the plant vacuole is of prime importance in buffering environmental perturbations and in coping with abiotic stress caused by drought, salinity, cold, or UV [67,68]. Sspon.07G0016410-2P is located in the chloroplast and is involved in post-transcriptional modification, which mediates the regulation of plant cold acclimation through complex mechanisms [69]. Sspon.04G0017410-4P is associated with carbohydrate transport and metabolism. In addition to the possible mechanisms mentioned above, transcriptome analysis results revealed that carbohydrate metabolism is coordinated with the degradation of amino acids to provide carbon skeletons to the tricarboxylic acid cycle. This coordination may help to maintain energetic balance during drought stress adaptation, facilitating recovery after the stress is alleviated [70]. Sspon.06G0005070-1A and Sspon.03G0012410-2B are involved in the regulation of nuclear chromatin structure. Alternative splicing (AS) can rapidly induce a large number of transcriptome changes to confer plant cold tolerance. Chromatin structure can regulate AS co-transcription, and DNA methylation can participate in *Arabidopsis* cold stress adaptation by regulating nucleosome proportion [71].

## 4. Materials and Methods

### 4.1. Treatment and Sampling

This experiment was conducted from March to November 2019 at the Sugarcane Research Institute, Guangxi Academy of Agricultural Sciences (GXAAS) in Nanning, Guangxi, China. Twenty-two genotypes of *S. spontaneum* selected from the 690 collections in the Guangxi Sugarcane Germplasm Nursery, GXAAS were planted in a sand bed for germination with single-bud setts. Upon growing to the three-leaf stage, the plants were transplanted to pots with 2–3 plants per pot. A complete randomized block design was applied with three replicates. The pot was 300 mm in height and 350 mm in diameter, filled with 17.5 kg of mixed soil (soil, organic fertilizer, and sand at 70:20:10, *w*/*w*). Holes were drilled into the bottom of the pot to enhance air permeability, and the pots were kept in an intelligent greenhouse for daily management. After 6 months of growth, the materials were divided into two groups: one group under normal temperature as the control group and the other group under low temperature. Treatment conditions were as follows: (1) normal-temperature control (CK), 25 °C, 250–300 µmol/m^2^·s light intensity, 12 h photoperiod, 60–70% relative humidity; (2) low-temperature treatment at 4 °C, with other conditions the same as the control. Samples were taken on the second, fourth, sixth, and eighth days, respectively. The leaves were collected at 8:00 a.m., encased with wet gauze, and brought to the laboratory for physiological and biochemical analysis. Three replicates per treatment were sampled for the measurement. Some of the samples were frozen in liquid nitrogen and stored at −80 °C for subsequent proteomic analysis.

### 4.2. Physiological and Biochemical Index Measurement

The content of SP was determined using the Coomassie brilliant blue G-250 method [72]; the content of SS was determined using the anthrone sulfate colorimetric method [73]; the content of MDA was determined using the thiobarbituric acid colorimetric method [74]; the content of Pro was determined using the sulfosalicylic acid method [75]; the activities of SOD [EC:1.15.1.1] and POD [EC:1.11.1.7] were determined using the nitro blue tetrazolium method [73] and the guaiacol method [76], respectively.

### 4.3. Data-Independent Acquisition (DIA)-Based Quantitative Proteomic Analysis

#### 4.3.1. Protein Extraction, Concentration Determination, and Enzymatic Hydrolysis

Liquid nitrogen was added to 0.1 g of leaf tissue in a precooled mortar; then, the sample was ground into powder. The powder was transferred to a 1.5 mL EP tube, followed by adding 0.5 mL of lysis buffer (containing 8 M urea, 2 mM EDTA, 10 mM DTT, 1% PMSF), and vortexing for 1 min to mix. After ultrasonication at 4 °C, the mixture was incubated on ice for 20 min. The supernatant was taken, and three volumes of 10% precooled TCA/acetone was added, before storage at −20 °C for 1–2 h. The supernatant was discarded after centrifugation, and the precipitate was washed with acetone reagent and then centrifuged. This step was repeated twice. The precipitate was placed on ice for 2–3 h, and then UT (8 M urea + 100 mM TEAB) was added for reconstitution, resulting in the protein solution. The modified Bradford protein detection kit was used to detect the protein concentration. According to the quantitative results, a 0.1 mg protein sample was taken, before adding DTT to regulate the concentration to 10 mM, and it was incubated at 37 °C for 30 min (reduction); IAM was added to make the final concentration 25 mM, and the solution was incubated for 20 min at room temperature in the dark for alkylation. After alkylation, 100 mM TEAB was added to reduce the concentration of urea in the protein solution to lower than 2 M before enzymatic hydrolysis. The enzymolysis steps were as follows: adding trypsin according to the mass ratio of 1:50, digesting overnight at 37 °C, and adding trypsin according to the mass ratio of 1:100, before performing a second enzymolysis at 37 °C for 4 h. The peptides after enzymatic hydrolysis were desalted (Ziptip C18, operated according to the product instructions) and lyophilized, before being reconstituted with 0.1% formic acid (FA); the peptide concentration was measured at a wavelength of 280 nm in a microspectrophotometer.

#### 4.3.2. HPLC Fractionation of Peptides

The lyophilized peptides were resuspended in liquid A (2% ACN, pH 10). The peptides were fractionated using high-pH reverse HPLC. The column was a Waters Bridge Peptide BEH C18 (130Å, 3.5 μm, 4.6 × 250 mm). The step gradient was 2–98% acetonitrile, pH 10.0, with a flow rate of 0.5 mL/min. Sixty fractions were separated in 88 min, and then the peptides were combined into 20 fractions. After being vacuum freeze-dried, the peptide fractions were finally desalted using a micro-chromatography column (Ziptip C18) according to the product instructions, and the products were vacuum freeze-dried and stored at −20 °C for subsequent mass spectrometry identification.

#### 4.3.3. High-Resolution LC–MS/MS Analysis

The peptide samples were dissolved in mobile phase A of liquid chromatography (0.1% FA). One microliter of the solution was used for injection through the sample column [Acclaim PepMap^®^100 C18, 3 μm, 100Å (75 μm × 2 cm)] and analytical column [Acclaim PepMap^®^ RSLC C18, 2 μm, 100Å (50 μm × 15 cm)], followed by entry into the Q Exactive HF combined mass spectrometer for identification after gradient elution.

The peptides were separated using an ultrahigh liquid-phase system. Mobile phase A was a 0.1% FA aqueous solution, and mobile phase B was an 80% acetonitrile solution containing 0.1% FA. The liquid phase gradient was as follows: 0–10 min, 5% B; 10–67 min, 5–45% B; 67–70 min, 45–90% B; 70–75 min, 90% B; 75–75.1 min, 90–5% B; 75.1–85 min, 5% B. The liquid phase flow rate was 400 nL/min, and mass spectrometry was performed after separation. The eluent was injected into the NSI ion source at 1.9 kV electrospray voltage for ionization, and then analyzed by tandem mass spectrometry (MS/MS) using an LTQ-Orbitrap Elite. The acquisition mode was data-independent (DIA), and the parameters were set as follows: MS1 full scan mode with scan range from 400 *m*/*z* to 1200 *m*/*z*, resolution of 45,000, automatic gain control (AGC) of 1 × 10^6^, maximum injection time of 35 ms. A total of 31 DIA windows were collected with a resolution of 15,000, AGC was set to 1 × 10^5^ (automatic maximum injection time), NCE was set to 28, and the analysis time was 85 min.

#### 4.3.4. Parallel Reaction Monitoring (PRM) Targeting Protein Verification

In order to verify the reliability of the proteomics results, PRM technology was used to verify the differentially abundant proteins. The experimental procedure referred to a previous study [77], and Skyline software was used to extract the peak area from the PRM mass spectrum data [78]. In this study, five DAPs were selected for verification by PRM.

### 4.4. Data Analysis

Physiological and biochemical index data were statistically analyzed using Excel 2007 and SPSS 15.0. The membership function method was used to evaluate and screen the cold tolerance ability of the tested genotypes by calculating the membership function value and the average membership degree.

The calculation formula of the membership function [4] value was as follows:

R (X_i_) = (X_i_ − X_min_)/(X_max_ − X_min_). (1)

The calculation formula of the anti-membership function was as follows:

R (Xi) = 1 − (X_i_ − X_min_)/(X_max_ − X_min_). (2)

X_i_ in the equations represents the measured value of the index, while X_max_ and X_min_, respectively represent the maximum and minimum values of a certain index in the measured material.

The raw data from the DIA proteomic analysis were processed and analyzed with Skyline v20.2.0.343 using default parameters. The target–decoy strategy was used to control the peptide false discovery rate (FDR) to less than 1%. After peak extraction and area calculation, the results were exported to a tabular format for further quantitative analysis. Disclosure of differentially abundant proteins was based on the following cutoff values: a twofold change and an FDR-adjusted *p*-value of 0.05. The DAPs were annotated through GO, KEGG, and COG/KOG databases to determine their functions.

## 5. Conclusions

**Figure 6:**
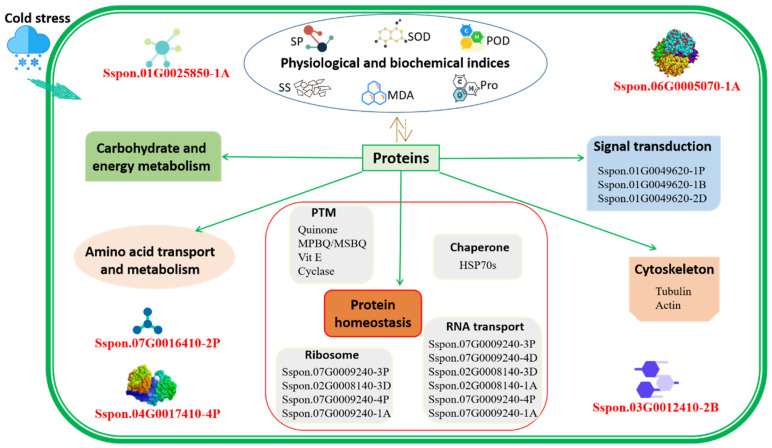
A putative regulatory network of *S. spontaneum* response to low temperature. Cold stress induces the variation of physiological and biochemical indices, as well as the abundance change in related proteins to coordinate a response. The proteins involved in cold stress response include those in pathways related to protein homeostasis, carbohydrate and energy metabolism, amino acid transport and metabolism, signal transduction, and the cytoskeleton. Five proteins identified for the first time in sugarcane are illustrated in red bold font. PTM, post-translational modification. SP, soluble protein. SS, soluble sugar. MDA, malondialdehyde. SOD, superoxide dismutase. POD, peroxidase.

This work classified the *S. spontaneum* germplasms into different groups on the basis of their physiological and biochemical indices using the membership function value method. The indices in the high-tolerance genotype 1027 and the low-tolerance genotype 3217 were consistent with their tolerance ability, hinting at the feasibility of the indices and the method in classifying the cold tolerance of sugarcane germplasms. According to the DIA-based proteomic analysis of the two genotypes with contrasting cold tolerance, the proteins associated with protein homeostasis, carbohydrate and energy metabolism, amino acid transport and metabolism, signal transduction, and the cytoskeleton may be involved in sugarcane’s tolerance to cold stress. This work not only promotes the elucidation of the mechanism underlying cold tolerance in sugarcane but also provides the germplasms and targets for breeding sugarcane varieties with cold tolerance via a conventional program and molecular technique. A putative regulatory network of sugarcane response to cold stress is proposed (Figure 6).

## Figures and Tables

**Table 1:** Cold tolerance classification of *S. spontaneum* according to membership function values based on physiological and biochemical indices.

Genotypes	Membership Function Value						Average	Cold Tolerance	Rank
	SP	SS	MDA	Pro	SOD	POD			
1027	0.528	0.242	0.943	0.940	1.000	0.906	0.760	HT	1
3411	0.904	1.000	0.882	0.561	0.464	0.426	0.706	HT	2
1016	0.658	0.300	0.834	1.000	0.327	0.422	0.590	MT	3
3405	0.793	0.357	0.678	0.000	0.646	1.000	0.579	MT	4
1015	0.981	0.457	0.612	0.116	0.296	0.545	0.501	MT	5
3420	0.946	0.099	0.826	0.540	0.253	0.206	0.478	MT	6
2805	0.883	0.231	0.744	0.481	0.076	0.395	0.468	MT	7
1006	0.493	0.308	1.000	0.073	0.116	0.614	0.434	MT	8
2605	0.975	0.192	0.000	0.809	0.149	0.479	0.434	MT	9
2806	0.586	0.559	0.812	0.353	0.044	0.229	0.430	MT	10
1316	1.000	0.152	0.335	0.069	0.208	0.798	0.427	MT	11
3413	0.587	0.225	0.801	0.497	0.077	0.374	0.427	MT	12
2601	0.763	0.266	0.639	0.156	0.196	0.517	0.423	MT	13
1208	0.354	0.676	0.732	0.017	0.147	0.537	0.411	MT	14
2812	0.372	0.592	0.506	0.524	0.136	0.301	0.405	MT	15
3316	0.763	0.104	0.678	0.290	0.185	0.398	0.403	MT	16
2809	0.524	0.524	0.549	0.364	0.141	0.314	0.403	MT	17
1009	0.000	0.198	0.848	0.521	0.260	0.482	0.385	MT	18
1493	0.648	0.217	0.796	0.230	0.062	0.289	0.374	LT	19
3416	0.517	0.138	0.982	0.521	0.066	0.000	0.371	LT	20
3703	0.765	0.259	0.499	0.205	0.004	0.052	0.297	LT	21
3217	0.272	0.000	0.993	0.067	0.000	0.341	0.279	LT	22

Note: HT, high tolerance; MT, medium tolerance; LT, low tolerance.

**Table 2:** PRM verification of DAPs.

Protein ID	Comparison	Fold Change (RPM)	Fold Change (DIA)
Sspon.03G0012410-2B	HT-CK vs. LT-CK	0.434	0.03731
Sspon.06G0005070-1A	HT-CK vs. LT-CK	0.433	0.03731
Sspon.07G0016410-2P	HT-T vs. LT-T	2.808	98.92528
Sspon.01G0025850-1A	LT-T vs. LT-CK	0.01	0.01165
Sspon.04G0017410-4P	HT-T vs. LT-T	1.68	28.5406

## Data Availability

The proteomic data reported in this paper have been deposited to the ProteomeXchange Consortium via the PRIDE [79] partner repository with the dataset identifier PXD038181 (accessed on 1 November 2022).

## Supplementary Materials

The following are available online at https://www.mdpi.com/article/10.3390/ijms232214244/s1.
