# Supplementary figures and images for: Cold-Induced Physiological and Biochemical Alternations and Proteomic Insight into the Response of Saccharum spontaneum to Low Temperature
Source: Int J Mol Sci. 2022 Nov 17;23(22):14244. doi: 10.3390/ijms232214244 (PMC9692960; doi:10.3390/ijms232214244)

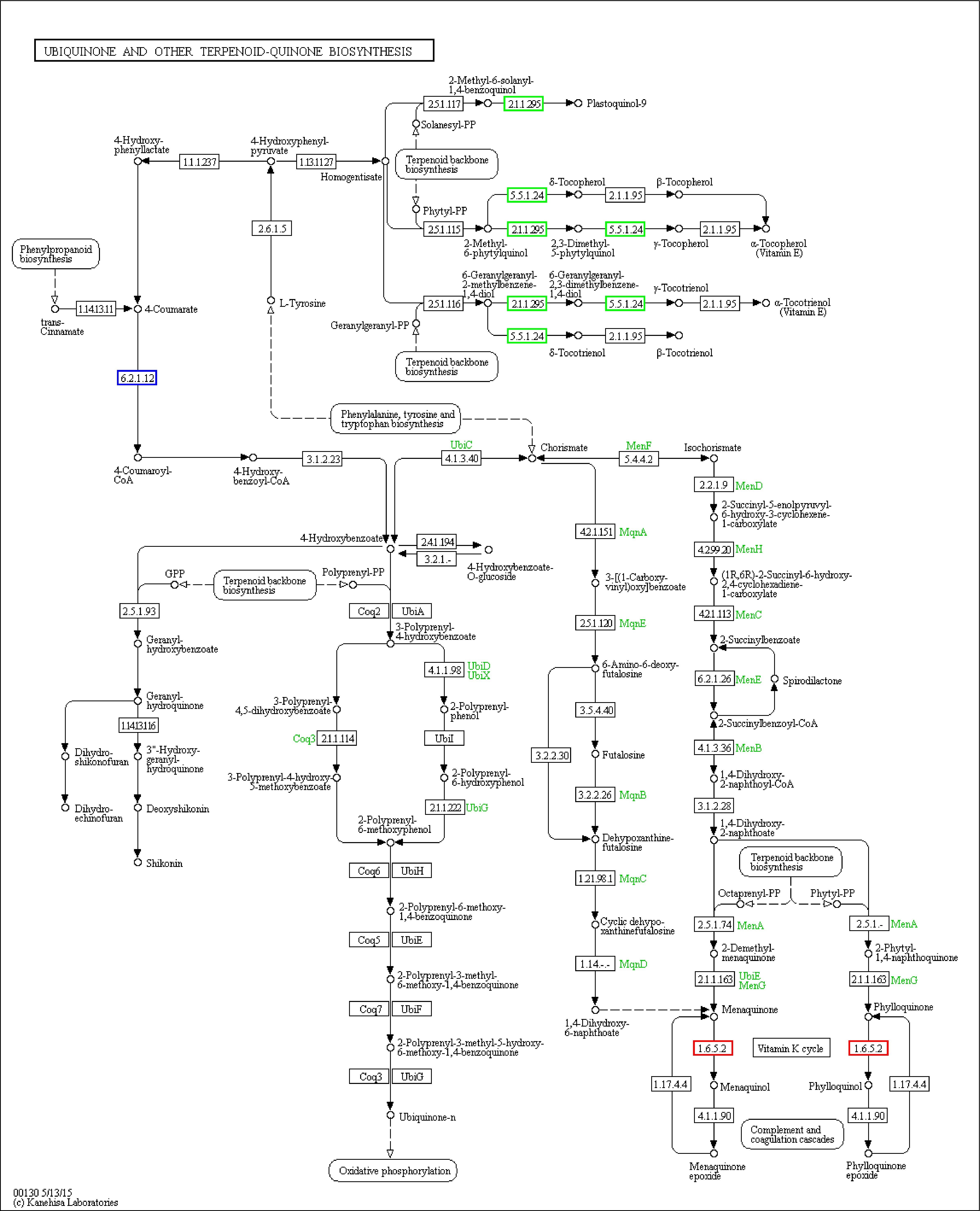

Supplement: Supplementary file 1 [file ijms-23-14244-s001.zip › ijms-2037950 supplementary/Figure S1. Ubiquitination-mediated protein degradation pathway.jpg]

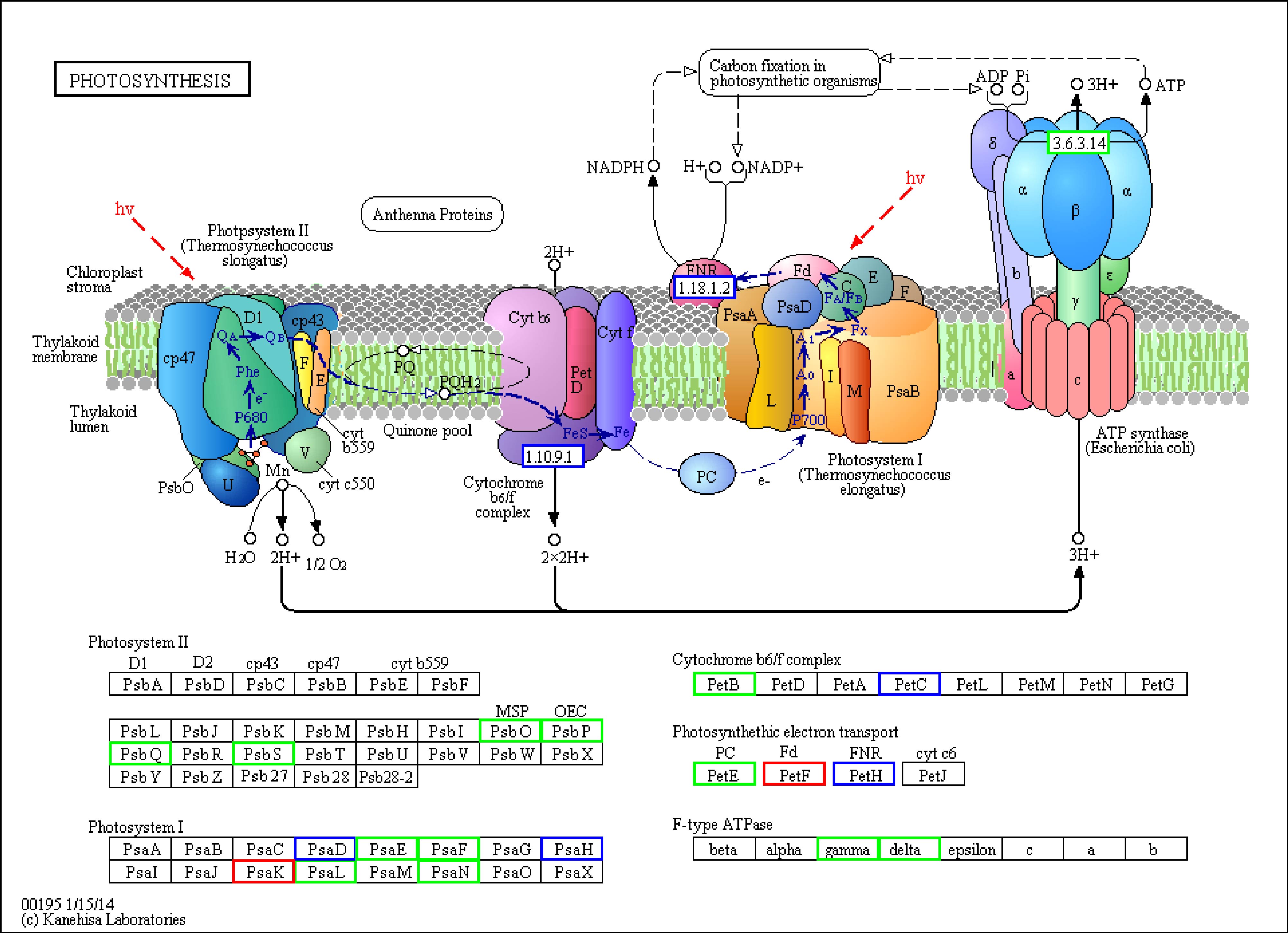

Supplement: Supplementary file 1 [file ijms-23-14244-s001.zip › ijms-2037950 supplementary/Figure S2. Photosynthesis pathway.jpg]

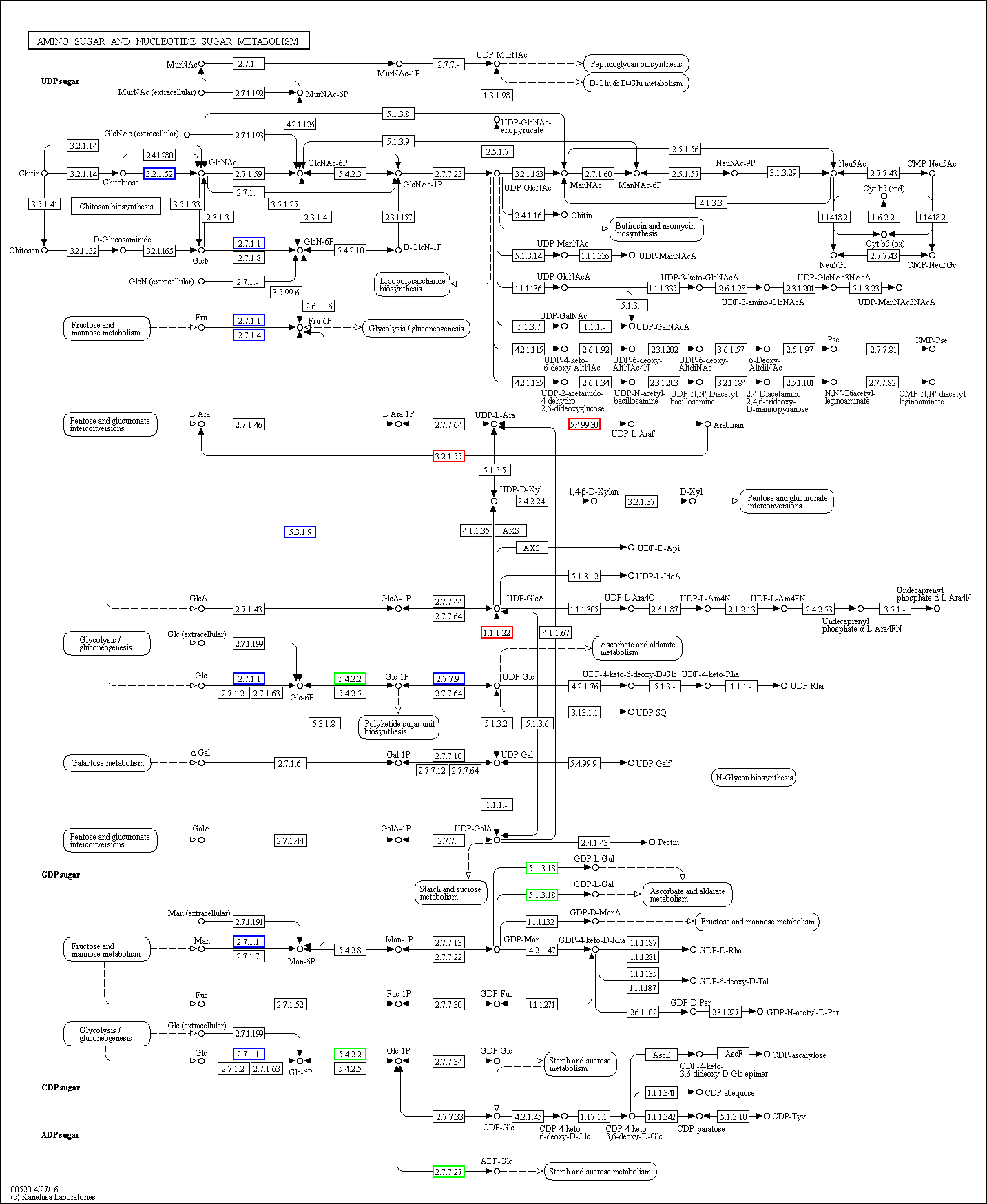

Supplement: Supplementary file 1 [file ijms-23-14244-s001.zip › ijms-2037950 supplementary/Figure S3. Amino sugar and nucleotide sugar metabolism pathway.png]
